# Colchicine prevents NSAID-induced small intestinal injury by inhibiting activation of the NLRP3 inflammasome

**DOI:** 10.1038/srep32587

**Published:** 2016-09-02

**Authors:** Koji Otani, Toshio Watanabe, Sunao Shimada, Shogo Takeda, Shigehiro Itani, Akira Higashimori, Yuji Nadatani, Yasuaki Nagami, Fumio Tanaka, Noriko Kamata, Hirokazu Yamagami, Tetsuya Tanigawa, Masatsugu Shiba, Kazunari Tominaga, Yasuhiro Fujiwara, Tetsuo Arakawa

**Affiliations:** 1 Department of Gastroenterology, Osaka City University Graduate School of Medicine 1-4-3 Asahimachi, Abeno-ku, Osaka, Japan

## Abstract

The inflammasome is a large, multiprotein complex that consists of a nucleotide-binding oligomerization domain-like receptor (NLR), an apoptosis-associated speck-like protein containing a caspase recruitment domain, and pro-caspase-1. Activation of the inflammasome results in cleavage of pro-caspase-1 into cleaved caspase-1, which promotes the processing of pro-interleukin (IL)-1β into mature IL-1β. We investigated the effects of colchicine on non-steroidal anti-inflammatory drug (NSAID)-induced small intestinal injury and activation of the NLR family pyrin domain-containing 3 (NLRP3) inflammasome. Colchicine treatment inhibited indomethacin-induced small intestinal injury by 86% (1 mg/kg) and 94% (3 mg/kg) as indicated by the lesion index 24 h after indomethacin administration. Colchicine inhibited the protein expression of cleaved caspase-1 and mature IL-1β, without affecting the mRNA expression of NLRP3 and IL-1β. Although treatment with recombinant IL-1β (0.1 μg/kg) did not change the severity of small intestinal damage, the preventive effects of colchicine were abolished by supplementation with the same dose of recombinant IL-1β. Indomethacin-induced small intestinal damage was reduced by 77%, as determined by the lesion index in *NLRP3*^−/−^ mice, and colchicine treatment failed to inhibit small intestinal damage in *NLRP3*^−/−^ mice. These results demonstrate that colchicine prevents NSAID-induced small intestinal injury by inhibiting activation of the NLRP3 inflammasome.

As the use of non-steroidal anti-inflammatory drug (NSAID) increases in an aging society, severe gastrointestinal injury and bleeding occur more frequently and have become a major problem. Several studies using video-capsule endoscopy have proven that NSAID can damage the small intestine as well as the upper gastrointestinal tract^1^,^2^,^3^,^4^. Although acid suppressants such as proton pump inhibitors (PPIs) and histamine H_2_-receptor antagonists prevent NSAID-induced damages to the upper gastrointestinal tract, these agents are not effective against small intestinal damage as hydrochloric acid is not produced in the small intestine and gastric acid is neutralized by bicarbonate in the duodenum^5^,^6^. Moreover, both human and experimental animal studies have raised the possibility that PPIs might exacerbate the damage^3^,^7^,^8^,^9^,^10^. There are no established treatments for NSAID-induced enteropathy at the present time. In this regard, understanding of the pathophysiology of NSAID-induced enteropathy is urgently required to enable the development of useful drugs.

During the development of NSAID-induced enteropathy, the small intestinal mucosa is exposed to various types of danger signals including pathogen-associated molecular patterns and damage-associated molecular patterns, and the innate immune response occurs in case these danger signals are recognized by pattern recognition receptors such as Toll-like receptors (TLRs) and nucleotide-binding oligomerization domain-like receptors (NLRs)^11^,^12^.

We previously demonstrated that the NSAID-triggered inflammatory response in the small intestine was TLR4-dependent. In addition to decreased production of prostaglandin due to cyclooxygenase inhibition, many factors play a crucial role in the onset of NSAID-induced intestinal damage, such as enterohepatic circulation, focal microvascular event, inducible nitric oxide synthase/nitric oxide, attenuation of phosphatidylcholine and surface hydrophobicity, disruption of surface membrane integrity, and mitochondrial malfunction^13^,^14^,^15^,^16^,^17^,^18^,^19^,^20^. Once the mucosal barrier function has been disrupted by these factors, luminal gram-negative bacteria can enter the epithelium, and lipopolysaccharide from these bacteria is then recognized by TLR4 on macrophages. In addition, high mobility group box 1, a nuclear protein regulating gene transcription, is released from injured epithelial cells and binds to TLR4, resulting in nuclear factor-κB (NF-κB) activation through the myeloid differentiation primary-response 88-dependent pathway. This induces the expression of multiple inflammatory cytokines including tumor necrosis factor (TNF)-α, and finally leads to small intestinal injury^21^,^22^.

On the other hand, NLRs are present inside the cell membrane and their activation by danger signals causes the formation of an inflammasome, a large multiprotein complex that consists of NLR, an apoptosis-associated speck-like protein containing a caspase recruitment domain (ASC), and pro-caspase-1. Activation of the inflammasome results in the cleavage of pro-caspase-1 into the cleaved form of caspase-1, which promotes the processing of pro-interleukin (IL)-1β into mature IL-1β, and leads to the induction and progression of the inflammatory response and cell death. NLR family, pyrin domain-containing 3 (NLRP3), also known as NALP3 or cryopyrin, is the most investigated molecule among the inflammasomes. We recently found that the NLRP3 inflammasome-derived IL-1β played a crucial role in NSAID-induced small intestinal damage^23^. IL-1β is a highly proinflammatory cytokine produced by macrophages and monocytes^24^. It plays a key role in the febrile response as an endogenous pyrogen and can also cause autoinflammatory diseases^25^.

The inflammasome was originally identified from genetic analyses of autoinflammatory diseases, a set of systemic inflammatory diseases caused by abnormalities of innate immunity without autoimmunity and infection^26^. Autoinflammatory diseases narrowly specify hereditary periodic fever syndrome such as cryopyrin-associated periodic syndrome, familial Mediterranean fever, and TNF receptor-associated periodic syndrome, and broadly include gout, pseudogout, Behçet’s disease, and Adult Still’s disease. It was recently reported that the NLRP3 inflammasome could be induced by monosodium urate or uric acid, the etiological agent of gouty attacks, and that colchicine, a therapeutic drug used for gout, suppressed the activation of the NLRP3 inflammasome and relieved gout^27^,^28^. In this study, we used a mouse model to investigate the effects of colchicine on NSAID-induced small intestinal injury and the activation of the NLRP3 inflammasome.

## Results

### Small intestinal damage and expression of inflammasome components after indomethacin administration

To induce small intestinal injury, non-fasted mice were administered 10 mg/kg indomethacin in a 0.5% carboxymethylcellulose solution by gavage, and were sacrificed 0, 3, 6, 12, and 24 h after indomethacin administration. A 1% solution of Evans blue was injected intravenously 30 min prior to sacrifice and the area (mm^2^) of macroscopically visible lesion was measured, summed per small intestine, and used as the lesion index.

**Figure 1:**
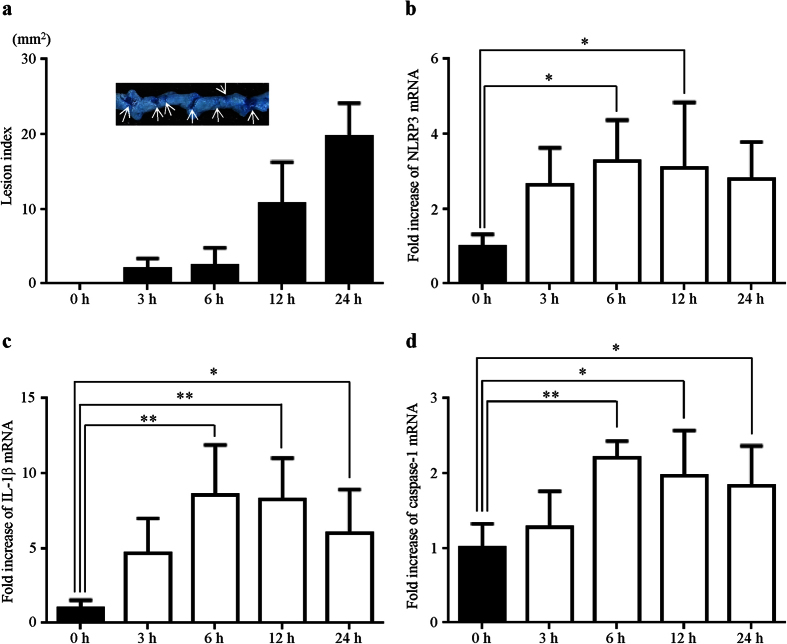
Small intestinal damage and expression of inflammasome components after indomethacin administration. (**a**) Time-course of changes in the lesion index after indomethacin administration. The lesion index was defined as the summed total area (mm^2^) of damaged tissue stained with Evans blue (arrows). (**b–d**) Time-courses of changes in NLRP3 (**b**) IL-1β (**c**) and caspase-1 (**d**) were determined using real-time RT-PCR. The mRNA levels are expressed as ratios of the mean value for vehicle-treated mice (0 h). n = 5**–**8; **P < 0.01, *P < 0.05.

We macroscopically confirmed that indomethacin induced multiple lesions in the small intestine indicated by dark blue staining with Evans after indomethacin administration. Small intestinal damage was macroscopically observed 3 h after indomethacin administration, and the lesion index increased in a time-dependent manner after indomethacin administration (Fig. 1a).

As we have previously reported that the NLRP3 inflammasome plays a crucial role in NSAID-induced small intestinal damage^23^, we examined the time-course for changes in NLRP3 and the other inflammasome components during the development of indomethacin-induced small intestinal damage using quantitative real-time RT-PCR. The mRNA levels of NLRP3 (Fig. 1b), IL-1β (Fig. 1c), and caspase-1 (Fig. 1d) in the small intestine significantly increased and peaked 6 h after indomethacin administration.

### Preventive effects of colchicine treatment on indomethacin-induced small intestinal injury

**Figure 2:**
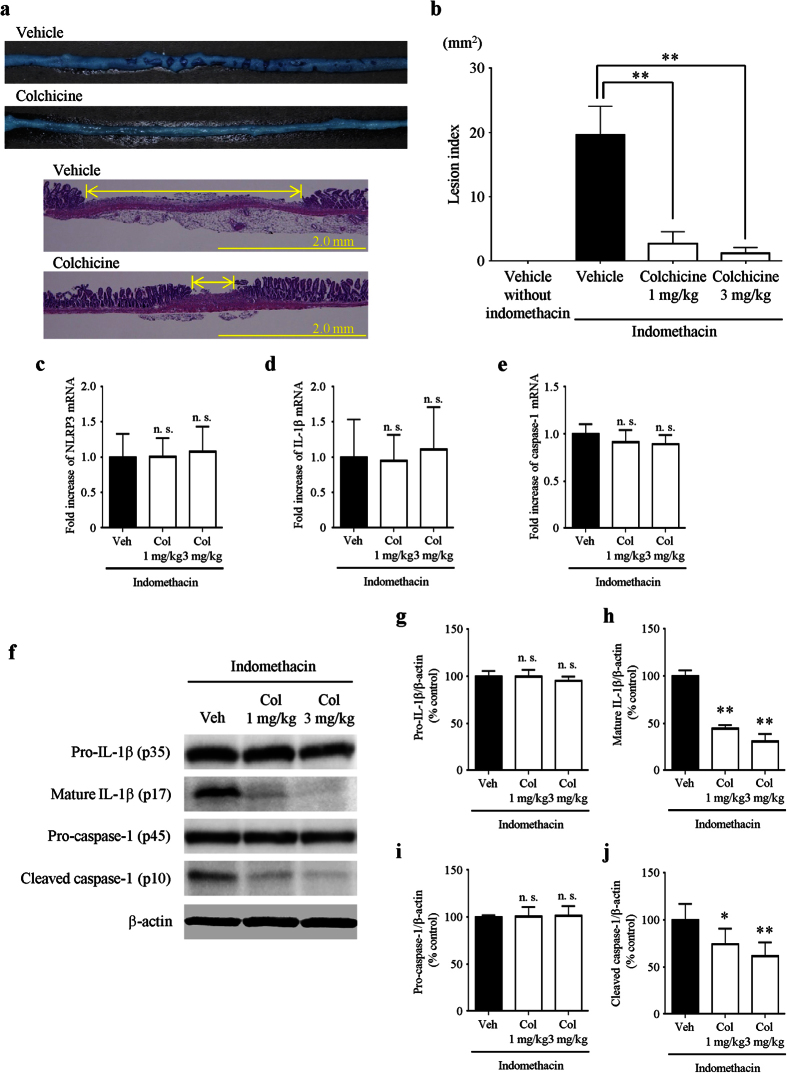
Preventive effects of colchicine treatment on indomethacin-induced small intestinal injury. (**a**) Representative images of damaged lesions stained with Evans blue (upper) and hematoxylin/eosin (lower). (**b**) The lesion index was defined as the summed total area of damaged tissue. (**c–e**) Expression of NLRP3 (**c**) IL-1β (**d**) and caspase-1 (**e**) was determined by real-time RT-PCR. The mRNA levels are expressed as ratios of the mean value for vehicle-treated mice. (**f–j**) Representative images from western blotting (**f**) and the relative expression of pro-IL-1β (**g**) mature IL-1β (**h**) pro-caspase-1 (**i**), and cleaved caspase-1 (**j**). The protein levels were normalized to β-actin. n = 5–8; **P < 0.01, *P < 0.05; n. s., not significant; Veh, vehicle; Col, colchicine.

We confirmed that vehicle treatment without indomethacin did not cause any damaged lesions in the small intestine in advance. Vehicle or colchicine (1 mg/kg) was administered orally 30 min prior to indomethacin administration. The lesions stained with Evans blue in the small intestine were smaller in colchicine-treated mice than those in vehicle-treated mice 24 h after indomethacin administration. In addition, histological examination showed that colchicine-treated mice had less mucosal inflammation and ulceration and a decrease in the size and numbers of lesions compared with vehicle-treated mice (Fig. 2a). Colchicine treatment significantly reduced the lesion index at doses of 1 mg/kg and 3 mg/kg (by 86% and 94%, respectively) compared with vehicle treatment (Fig. 2b).

We next investigated the effect of colchicine on the mRNA levels of inflammasome components 6 h after indomethacin administration. In comparison with vehicle treatment, colchicine treatment did not significantly change the mRNA levels of NLRP3 (Fig. 2c), IL-1β (Fig. 2d), or caspase-1 (Fig. 2e) at doses of 1 mg/kg and 3 mg/kg.

Furthermore, we examined the effect of colchicine treatment on the protein expression of mature IL-1β (p17) and cleaved caspase-1 (p10) 6 h after indomethacin administration. Colchicine treatment significantly inhibited the protein levels of mature IL-1β at doses of 1 mg/kg and 3 mg/kg (by 56% and 69%, respectively) without affecting those of pro-IL-1β. Colchicine treatment also significantly inhibited the protein levels of cleaved caspase-1 at doses of 1 mg/kg and 3 mg/kg (by 26% and 39%, respectively) without affecting those of pro-caspase-1 (Fig. 2f–j).

### Preventive effect of colchicine treatment on the expression and localization of cleaved caspase-1 in the damaged small intestine

**Figure 3:**
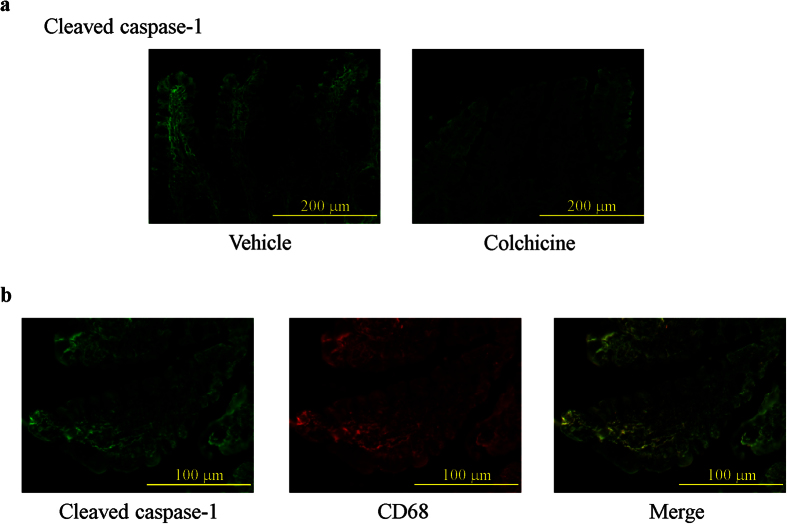
Preventive effect of colchicine treatment on the expression and localization of cleaved caspase-1 in the damaged small intestine. (**a**) Immunofluorescence detection of cleaved caspase-1 in vehicle-treated mice and colchicine-treated mice. (**b**) Co-localization of cleaved caspase-1 with CD68.

The localization and expression levels of cleaved caspase-1 protein were determined using immunofluorescence, which showed that cleaved caspase-1 was diffusely expressed in the lamina propria of the small intestinal mucosa 6 h after indomethacin administration, while mice treated with colchicine exhibited a marked decrease in cleaved caspase-1 expression (Fig. 3a). Double staining of cleaved caspase-1 with CD68 demonstrated that the majority of cells expressing cleaved caspase-1 were macrophages and monocytes (Fig. 3b).

### Effect of exogenous IL-1β and colchicine treatment on indomethacin-induced small intestinal injury

**Figure 4:**
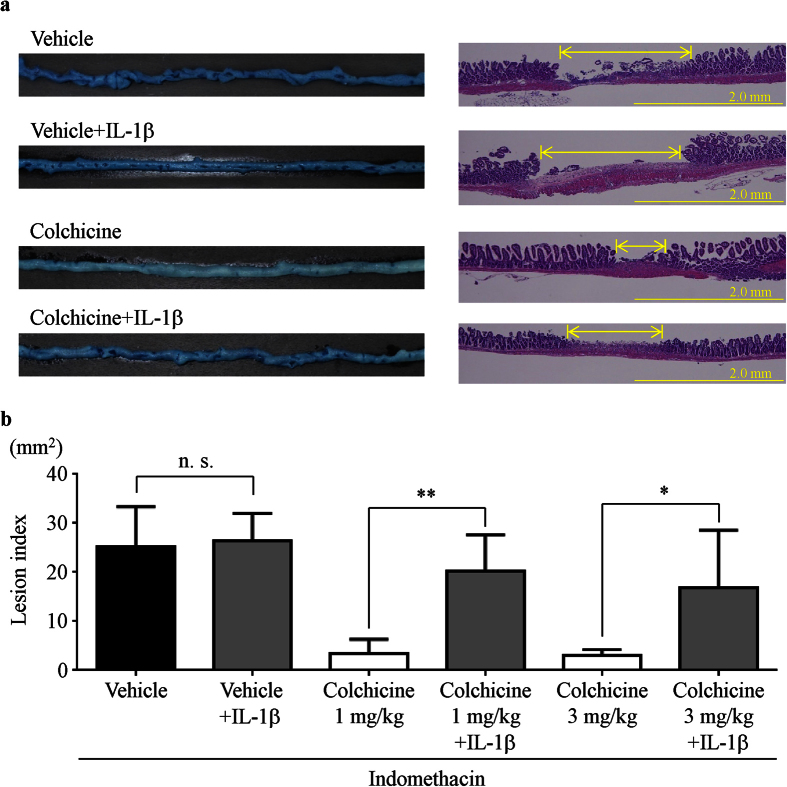
Effect of exogenous IL-1β and colchicine treatment on indomethacin-induced small intestinal injury. (**a**) Representative images of lesions stained with Evans blue (left) and hematoxylin/eosin (right). (**b**) The lesion index was defined as the summed total area of damaged tissue. n = 5**–**8; **P < 0.01, *P < 0.05; n. s., not significant.

To investigate the role of IL-1β in the development of NSAID-induced small intestinal damage, mice received vehicle or intraperitoneal injections of murine recombinant IL-1β (0.1 μg/kg) 3 h after indomethacin treatment. The administration of recombinant IL-1β prior to the indomethacin challenge abolished the preventive effects of colchicine against indomethacin-induced damage in both macroscopic (i.e., lesion index) and microscopic evaluations, but IL-1β supplementation did not affect the severity of damage in vehicle-treated mice (Fig. 4a,b).

### Preventive effects of colchicine treatment are mediated by suppression of the NLRP3 inflammasome

**Figure 5:**
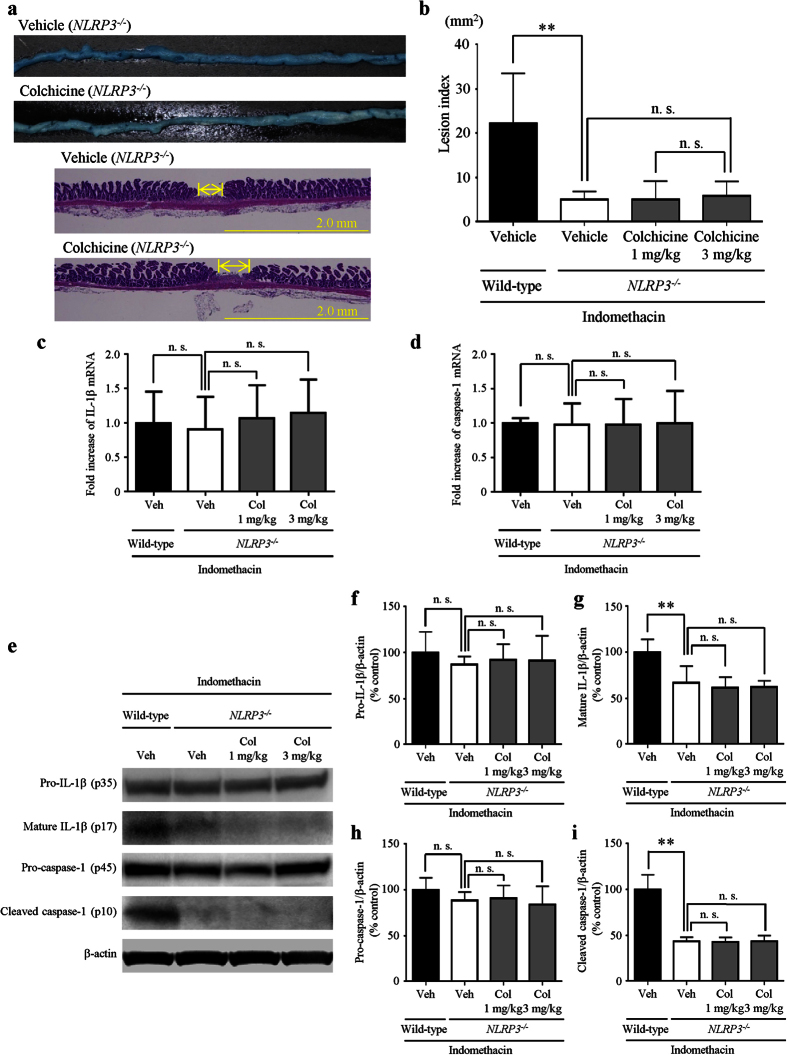
Preventive effects of colchicine treatment are mediated by suppression of the NLRP3 inflammasome. (**a**) Representative images of damaged lesions stained with Evans blue (upper) and hematoxylin/eosin (lower). (**b**) The lesion index was defined as the summed total area of damaged tissue. (**c,d**) Expression of IL-1β (**c**) and caspase-1 (**d**) was determined by real-time RT-PCR. The mRNA levels are expressed as ratios of the mean value for vehicle-treated wild-type mice. (**e–i**) Representative images of western blotting (**e**) and expression of pro-IL-1β (**f**) mature IL-1β (**g**) pro-caspase-1 (**h**) and cleaved caspase-1 (**i**). The protein levels were normalized to β-actin. n = 5**–**8; **P < 0.01; n. s., not significant; Veh, vehicle; Col, colchicine.

To confirm the involvement of the NLRP3 inflammasome/caspase-1/IL-1β axis in the suppressive effects of colchicine, vehicle or colchicine (1 or 3 mg/kg) was administered to mice with a genetic disruption of NLRP3 (*NLRP3*^−/−^ mice) before the administration of indomethacin. Consistent with a previous study^23^, indomethacin-induced small intestinal damage was macroscopically slight in *NLRP3*^−/−^ mice. Colchicine treatment did not further inhibit small intestinal damage in *NLRP3*^−/−^ mice (Fig. 5a). The indomethacin-induced small intestinal damage assessed using the lesion index was significantly reduced by 77% in *NLRP3*^−/−^ mice compared with that in wild-type mice, and we confirmed that colchicine treatment at doses of 1 mg/kg and 3 mg/kg failed to inhibit small intestinal damage in *NLRP3*^−/−^ mice (Fig. 5b).

We investigated the effects of colchicine treatment (1 mg/kg and 3 mg/kg) on the mRNA levels of inflammasome components in *NLRP3*^−/−^ mice. The mRNA levels of IL-1β (Fig. 5c) and caspase-1 (Fig. 5d) were not significantly altered in *NLRP3*^−/−^ mice compared with those in wild-type mice. In *NLRP3*^−/−^ mice, and colchicine treatment at doses of 1 mg/kg and 3 mg/kg did not significantly change the mRNA levels of IL-1β (Fig. 5c) and caspase-1 (Fig. 5d) compared with vehicle treatment.

We examined the effects of colchicine treatment on the protein expression of mature IL-1β (p17) and cleaved caspase-1 (p10) in *NLRP3*^−/−^ mice. The protein levels of mature IL-1β and cleaved caspase-1 were significantly reduced by 33% and 57%, respectively, in *NLRP3*^−/−^ mice compared with wild-type mice. The protein levels of pro-IL-1β and pro-caspase-1 were not changed in *NLRP3*^−/−^ mice compared with wild-type mice. The expression levels of these proteins were similar in *NLRP3*^−/−^ mice with and without colchicine treatment (Fig. 5e–i).

## Discussion

In this study, we examined the effect of colchicine treatment on NSAID-induced small intestinal injury and demonstrated that colchicine treatment prevented injury by inhibiting the activation of the NLRP3 inflammasome.

Colchicine treatment suppressed increases in the protein expression of cleaved caspase-1 and mature IL-1β without affecting the protein expression of pro-caspase-1 and pro-IL-1β or the mRNA expression of inflammasome components, suggesting that colchicine inhibited the cleavage of caspase-1 and the maturation processing of IL-1β. Administration of a low dose of exogenous IL-1β, which did not affect the severity of indomethacin-induced small intestinal damage, abolished the preventive effects of colchicine treatment on intestinal damage. This proves that the attenuation of small intestinal injury by colchicine is due to a deficiency of the mature form of IL-1β. Furthermore, we used *NLRP3*^−/−^ mice to examine whether colchicine inhibited the maturation processing of IL-1β by suppressing the activation of the NLRP3 inflammasome. *NLRP3*^−/−^ mice exhibited less severe damage compared with wild-type mice and colchicine did not further inhibit small intestinal damage in *NLRP3*^−/−^ mice. Collectively, these findings clearly indicate that the preventive effects of colchicine in indomethacin-induced small intestinal injury were mediated through the inhibition of NLRP3 inflammasome activation. To the best of our knowledge, this is the first report demonstrating that colchicine is an effective treatment for NSAID-induced enteropathy and acts by suppressing the activation of the NLRP3 inflammasome.

Colchicine is a type of alkaloid derived from *Colchicum autumnale* that has long been used as an effective treatment for gouty arthritis. In addition to gout, it has become evident that colchicine is also effective for treating familial Mediterranean fever^29^,^30^, Behçet’s disease^31^,^32^, Sweet’s disease^33^, palmoplantar pustulosis^34^,^35^, scleroderma^36^, and primary biliary cirrhosis^37^. Although colchicine was thought to act by inhibiting microtubule formation and cell division^38^, its anti-inflammatory effects were unclear. Martinon *et al*. reported that uric acid crystals did not induce IL-1β maturation in macrophages derived from *NLRP3*^−/−^ mice and neutrophil influx in response to uric acid crystals was reduced in mice deficient in NLRP3 inflammasome components^27^. Their findings revealed that uric acid crystals could activate the NLRP3 inflammasome, which resulted in IL-1β maturation and the induction of inflammatory responses. Misawa *et al*. recently reported that microtubules mediated the activation of the NLRP3 inflammasome and that acetylated α-tubulin mediated mitochondrial transport along microtubules^28^. If macrophages engulf uric acid crystals, the endolysosomal components leak into the cytoplasm and cause mitochondrial damage which can lead to the accumulation of acetylated α-tubulin. The authors suggested that acetylated α-tubulin mediated dynein-dependent transport of mitochondria along microtubules and the subsequent approximation of ASC to NLRP3 on the endoplasmic reticulum. Notably, colchicine suppressed the dynein-dependent transport and NLRP3 inflammasome activation.

It is known that two signals are required for NLRP3 inflammasome activation. The binding of pathogen-associated molecular patterns to TLRs or the binding of TNF-α and IL-1β to their corresponding receptors initiates the first signal (Signal 1) and pro-IL-1β is synthesized at the transcriptional level during this process. The second signal (Signal 2) is provided by danger signals such as extracellular adenosine triphosphate (ATP), bacterial toxins, amyloid β aggregates, uric acid crystals, cholesterol crystals, asbestos, or silica, and these activate the NLRP3 inflammasome, resulting in the cleavage of caspase-1 and the processing of IL-1β^39^,^40^,^41^,^42^,^43^. In NSAID-induced small intestinal injury, we have demonstrated that TLR4 signaling triggers NF-κB-mediated upregulation of NLRP3 and IL-1β mRNA (Signal 1)^21^,^23^. Subsequently, K^+^ efflux caused by the binding of extracellular ATP to the P2X_7_ receptor, the production of intracellular reactive oxygen species by mitochondria, and lysosomal enzymes induce the activation of the NLRP3 inflammasome, which results in the release of mature IL-1β (Signal 2)^12^,^37^,^44^. As colchicine markedly inhibited the protein expression of cleaved caspase-1 and mature IL-1β without preventing increases in the mRNA levels of NLRP3 and IL-1β, these results suggest that colchicine did not affect mechanisms involving Signal 1 in the inflammasome activation pathways, but inhibited NLRP3 inflammasome activation by suppressing the assembly of inflammasome components along microtubules in Signal 2. There have been reports that colchicine has various kinds of anti-inflammatory actions such as modulating the production of chemokines and prostanoids, inhibiting neutrophil and endothelial cell adhesion molecules, and eventually decreasing neutrophil degranulation, chemotaxis, and phagocytosis^45^,^46^. However, we found that colchicine failed to further inhibit small intestinal damage in *NLRP3*^−/−^ mice, suggesting that the inhibitory actions of colchicine might be mediated mainly through the inhibition of NLRP3 inflammasome activation.

In conclusion, colchicine prevents NSAID-induced small intestinal injury by inhibiting caspase-1 activation and IL-1β maturation that precede the activation of the NLRP3 inflammasome. As colchicine has been used for treating many patients with gout and other diseases, its safety and tolerability have already been established. A clinical trial should be urgently performed to prove the efficacy of colchicine for treating NSAID-induced small intestinal injury.

## Methods

### Animals

Specific-pathogen-free 8-week-old male mice were used. Wild-type C57BL/6 mice were purchased from Charles River Japan Inc. (Atsugi, Japan), and *NLRP3*^−/−^ mice on a C57BL/6 background were purchased from the Jackson Laboratory (Bar Harbor, ME). All mice were housed in polycarbonate cages with paper-chip bedding in an air-conditioned biohazard room with a 12-h light/12-h dark cycle. All animals had free access to food and water.

All experiments were carried out under the control of animal research committee in accordance with The Guidelines on Animal Experiments in Osaka City University Graduate School of Medicine, Japanese Government Animal Protection and Management Law (No. 105), and Japanese Government Notification on Feeding and Safekeeping of Animals (No. 6). All experimental procedures were approved by the Animal Care Committee of Osaka City University Graduate School of Medicine (Approval number 14031). All surgeries were performed under isoflurane, and all efforts were made to minimize suffering.

### Experimental design

To induce small intestinal injury, non-fasted mice were administered 10 mg/kg indomethacin (Sigma-Aldrich Co., St. Louis, MO) in a 0.5% carboxymethylcellulose solution by gavage, and were sacrificed 0, 3, 6, 12, and 24 h after indomethacin administration. To delineate the damage, 1% Evans blue was injected intravenously 30 min prior to sacrifice. The small intestine was opened along the anti-mesenteric attachment and examined for damage under a dissecting microscope with square grids (×10). The area (mm^2^) of macroscopically visible lesion was measured, summed per small intestine, and designated as the lesion index. To avoid the influence of Evans blue on the tissues used for real-time RT-PCR, the experiment for the lesion index and the experiment for mRNA expression of inflammasome components were performed separately.

To examine the effects of colchicine on NSAID-induced small intestinal injury, vehicle or colchicine (1 or 3 mg/kg; Wako Pure Chemical Industries, Ltd., Kyoto, Japan) was administered orally 30 min prior to indomethacin administration. Mice received intraperitoneal injections of sterilized phosphate buffered saline or mouse recombinant IL-1β (0.1 μg/kg; R&D Systems, Inc., Minneapolis, MN) 3 h after indomethacin treatment. Vehicle or colchicine (1 or 3 mg/kg) was also administered to *NLRP3*^−/−^ mice before indomethacin administration. We evaluated the lesion index 24 h after indomethacin administration, and examined mRNA and protein expression of inflammasome components 6 h after indomethacin administration.

### RNA isolation and determination of the mRNA expression levels of inflammatory cytokines and inflammasome components in small intestinal tissue

Total RNA was isolated from intestinal tissue using an ISOGEN kit (Nippon Gene Co., Ltd., Tokyo, Japan) according to manufacturer protocol. Complementary DNA was produced using the High Capacity RNA-to-cDNA Kit (Thermo Fisher Scientific Inc., MA) according to manufacturer protocol. Real-time quantitative RT-PCR analyses were performed using an Applied Biosystems 7500 Fast Real-Time PCR system and software (Thermo Fisher Scientific Inc.). The reaction mixture was prepared using the TaqMan Fast Universal PCR master mixture (Thermo Fisher Scientific Inc.) according to manufacturer protocol. Thermal cycling conditions were as follows: 45 cycles of 95 °C for 15 s and 60 °C for 1 min. The expression levels of NLRP3, IL-1β, and caspase-1 in small intestinal tissue were quantified using real-time RT-PCR and standardized to TaqMan glyceraldehyde-3-phosphate dehydrogenase (GAPDH; Thermo Fisher Scientific Inc.). The mRNA levels are expressed as ratios of the mean value for vehicle-treated mice. The primers and probes used for real-time RT-PCR are listed in Table 1.

### Western blot analysis

Small intestinal tissues were homogenized and lysed on ice in a buffer containing 0.5% NP-40, 40 mM Tris-HCl (pH 8.0), 120 mM NaCl, phosphatase inhibitor cocktail (PhosSTOP; Roche Applied Science, Indianapolis, IN), and a protease inhibitor cocktail (Complete Mini; Thermo Fisher Scientific Inc.). Protein levels in the lysate were measured using the modified bicinchoninic acid method. Proteins were denatured in sodium dodecyl sulfate sample buffer at 95 °C for 5 min and then subjected to 15% SDS-polyacrylamide gel electrophoresis and transferred to a PVDF membrane. Membranes were blocked in Tris-buffered saline (10 mM Tris-HCl, pH 7.5, 100 mM NaCl, 0.1% Tween-20) containing 5% skim milk and then incubated overnight with one of the following antibodies: goat polyclonal anti-IL-1β/IL-1F2 (diluted 1:500; AF-401-NA; R&D Systems, Inc.), rabbit polyclonal anti-caspase-1 (diluted 1:200; sc-514; Santa Cruz Biotechnology, Inc., Santa Cruz, CA), and mouse anti-β-actin (Sigma-Aldrich Co.). Bound antigen-antibody complexes were detected using the appropriate HRP-conjugated secondary antibodies and enhanced chemiluminescence according to manufacturer instructions (Amersham, Arlington Heights, IL). Relevant bands were quantified using laser-scanning densitometry and the expression level of each protein was normalized to that of β-actin. All data are expressed as percent changes from control tissues treated with vehicle before indomethacin administration.

### Histological and immunofluorescence studies

Tissue samples were fixed with 0.1 M phosphate buffer (PB, pH 7.4) containing 4% paraformaldehyde and embedded in Tissue-Tek OCT Compound (Sakura Finetek Japan, Tokyo, Japan). Serial 5-μm-thick cryostat sections were mounted on silanized slides (Dako, Tokyo, Japan). The tissue samples were incubated overnight at 4 °C with the following primary antibodies: goat polyclonal anti-cleaved caspase-1 p10 (diluted 1:50; sc-22166; Santa Cruz Biotechnology, Inc.) and rat monoclonal anti-CD68 antibody (diluted 1:200; ab53444; Abcam, Cambridge, MA). The primary antibodies were reacted with the corresponding secondary fluorescent-dye-conjugated antibodies: Alexa Fluor 488^®^ donkey anti-goat IgG (Thermo Fisher Scientific Inc.) or Alexa Fluor 594^®^ donkey anti-rat IgG (Thermo Fisher Scientific Inc.). The specimens were examined using a confocal microscope equipped with argon and argon-krypton laser sources.

### Statistical analysis

Values are expressed as mean ± standard deviation. An independent Student’s t-test was used to compare differences between two groups. One-way analysis of variance was used to compare differences between multiple groups, and the results were analyzed by Tukey’s multiple comparisons test. *P*-values < 0.05 were considered to be statistically significant.

## Additional Information

**How to cite this article**: Otani, K. *et al*. Colchicine prevents NSAID-induced small intestinal injury by inhibiting activation of the NLRP3 inflammasome. *Sci. Rep*. **6**, 32587; doi: 10.1038/srep32587 (2016).

## Figures and Tables

**Table 1:** PCR primers and TaqMan probes.

Gene	Primers and probes	
NLRP3	Primer (forward)	5′-TGCCTTGGGAGACTCAGGAG-3′
	Primer (reverse)	5′-CAGAGGTCAGAGCTGAACAACA-3′
	Probe	5′-FAM-AGGCCGGAATTCACCAACCCCAGCT-TAMRA-3′
IL-1β	Primer (forward)	5′-ACAGGCTCCGAGATGAACAAC-3′
	Primer (reverse)	5′-CCATTGAGGTGGAGAGCTTTC-3′
	Probe	5′-FAM-GAAAAAGCCTCGTGCTGTCGGACCCATAT-TAMRA-3′
Caspase-1	Primer (forward)	5′-GGACATCCTTCATCCTCAGAAACA-3′
	Primer (reverse)	5′-TTTCTTTCCATAACTTCTGGGCTTT-3′
	Probe	5′-FAM-TGGCACATTTCCAGGACTGACTGGGACC-TAMRA-3′
